# Protocol to train a support vector machine for the automatic curation of bacterial cell detections in microscopy images

**DOI:** 10.1016/j.xpro.2024.102868

**Published:** 2024-02-02

**Authors:** Bart Steemans, Sander K. Govers

**Affiliations:** 1 Department of Biology, KU Leuven, 3001 Leuven, Belgium

**Keywords:** Microbiology, Microscopy, Systems biology

## Abstract

Manual curation of bacterial cell detections in microscopy images remains a time-consuming and laborious task. This work offers a comprehensive, step-by-step tutorial on training a support vector machine to autonomously distinguish between good and bad cell detections. Jupyter notebooks are included to perform feature extraction, labeling, and training of the machine learning model. This method can readily be incorporated into profiling pipelines aimed at extracting a multitude of features across large collections of individual cells, strains, and species.

For complete details on the use and execution of this protocol, please refer to Govers et al.^1^

## Highlights

• Protocol for training an SVM model to distinguish good from bad cell detections
• Ready-to-use Jupyter notebooks for feature extraction, labeling, and training
• Experimental procedure for generating microscopy images of bacterial cells

## Abstract (editor highlights)

Publisher’s note: Undertaking any experimental protocol requires adherence to local institutional guidelines for laboratory safety and ethics.

## Graphical abstract

**Figure:**
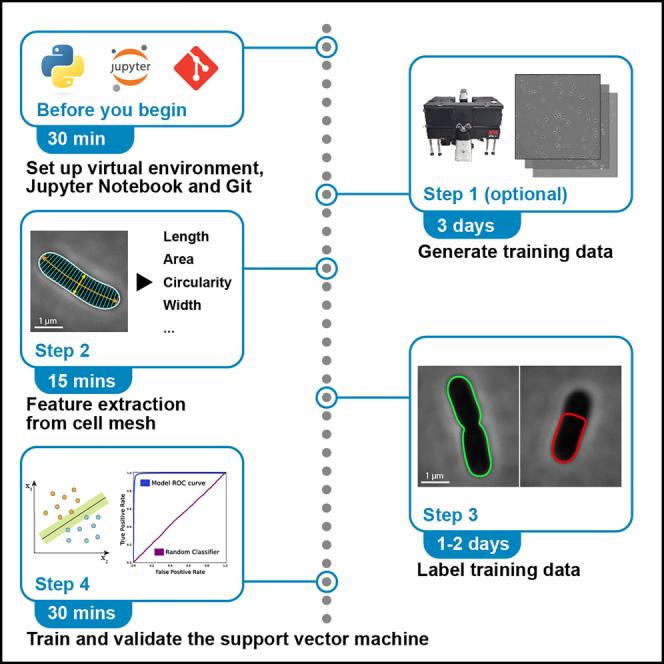

## Before you begin

The protocol below details the generation of training data and the training of a support vector machine (SVM) model for the curation of *Escherichia coli* cell detections in phase contrast, snapshot microscopy images. However, the protocol can be readily used to train SVM models for cell detections of other bacterial species or even mixtures of species. In principle, this approach can even be extended beyond bacterial cells to yeast, plant or human cells. This will likely require customization of the feature extraction to accommodate different cell types, data structures and imaging approaches, but training of the SVM model would occur in the exact same way.

### Setting up the python environment

**Timing: 30 min**

Before any training can be performed using the provided Jupyter notebooks, it is important to install and set up the required Python environment.

1. Install Anaconda:
  a. Download the appropriate version of Anaconda for your operating system from the official website (https://www.anaconda.com/products/individual).
  b. Follow the installation instructions specific to your operating system.

**CRITICAL:** This protocol is tailored for Windows operating systems.

2. Create a Virtual Environment (troubleshooting 1):
  a. Open an Anaconda Prompt/Terminal.
  b. Create a new virtual environment by running the following code:

> conda create --name svm_env python=3.10.8

When asked ‘Proceed ([y]/n)?’, answer y.

***Note:*** Python version 3.10.8 is used in all our analyses.

3. Activate the Virtual Environment and install the required libraries:
  ***Note:*** Run all code in the Anaconda Prompt unless specified otherwise.
  a. Activate the environment by running this line of code:
    > conda activate svm_env
  b. Run the following lines of code separately to install the required libraries:
    >pip install numpy==1.23.4
    >pip install pandas==2.0.1
    >pip install tifffile==2022.10.10
    >pip install tqdm==4.64.1
    >pip install opencv-python-headless==4.6.0.66
    >pip install scikit-image==0.19.3
    >pip install scipy==1.9.3
    >pip install shapely==2.0.1
    >pip install scikit-learn==1.1.3
    >pip install matplotlib==3.6.2
    ***Note:*** We used these library versions for writing the notebooks. Newer versions might no longer be compatible with each other and prevent the notebooks from working correctly.
4. Set up Jupyter Notebook:
  a. In the Python environment, install Jupyter Notebook by running the following code:
    > conda install jupyter
    When asked ‘Proceed ([y]/n)?’, answer y.
  b. Launch Jupyter Notebook by running the following code:
    > jupyter notebook
  c. Jupyter Notebook should open in your default web browser, displaying the file browser interface.
5. Clone our GitHub repository using Git:
  a. Download Git from the official website: https://git-scm.com/downloads.
  b. Open a Command Prompt (not the Anaconda Prompt).
  c. Run the following code to clone our GitHub Repository:
    > git clonehttps://github.com/Govers-Lab/Steemans_and_Govers_2023.git.
  d. The Steemans_and_Govers_2023 repository that contains folders with the notebooks, feature data and labeled feature data is now downloaded on your local computer and located in your current working directory.
  e. For immediate testing of the notebooks, readers are referred to the ‘feature extraction’ section.

### Data structure: cell mesh

**Figure 1:**
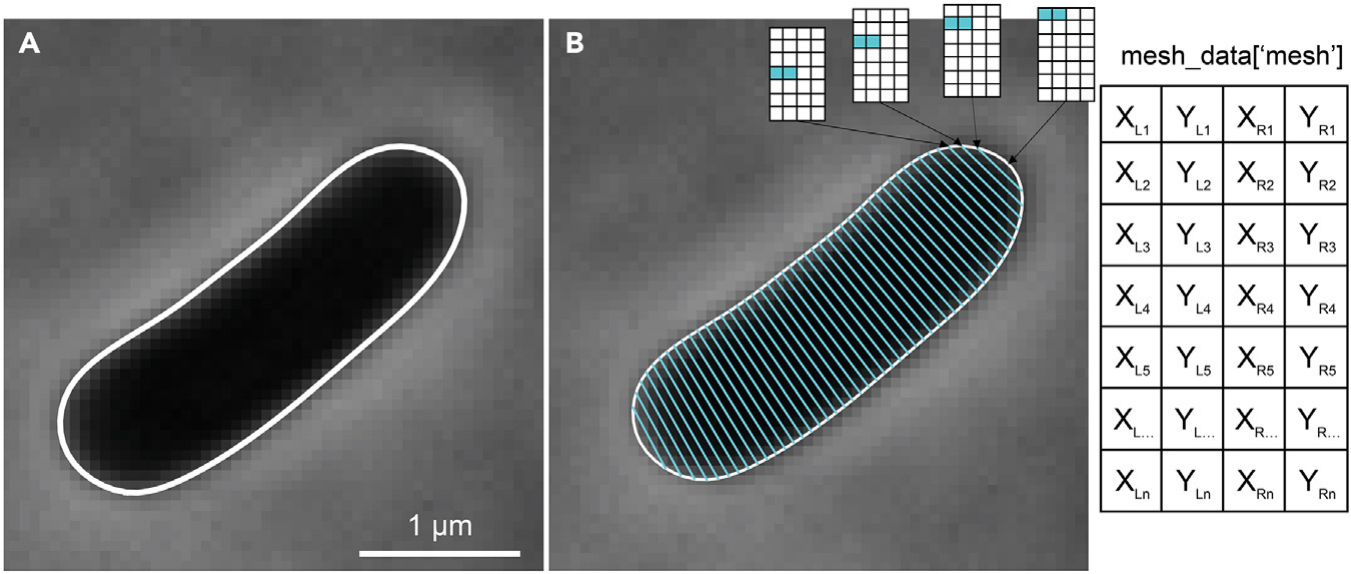
Cell contours and the cell mesh structure (A) Phase contrast image of a cell and its subpixel contour (white). (B) Visualization of the cell mesh (cyan). The first two columns of the nx4 matrix correspond to the coordinates of the left side of the cell and the last two columns represent the coordinates of the right side of the cell.

An SVM to curate cell detections can be trained using relevant, quantifiable, and representative cellular features that are extracted from cell contours. A cell contour delineates the outline of an individual cell and is obtained by segmenting raw microscopy images (Figure 1A). Several software packages and (deep neural network) image-segmentation algorithms currently exist that enable the identification of individual bacterial cells in phase contrast images.^2^^,^^3^^,^^4^^,^^5^^,^^6^ For this protocol, we start from the cell mesh structure that is obtained by using Oufti,^7^ an existing open-source software package for the analysis of bacterial microscopy data. This cell mesh consists of an nx4 matrix, in which the first two columns represent the x and y coordinates of the left side of the cell and the last two columns those of the right side of the cell (Figure 1B). The first row corresponds to a cell pole, the subsequent rows to corresponding points along each side of the cell until the last row, which corresponds to the other cell pole (Figure 1B). Cell contour coordinates obtained by any segmentation algorithm or software can be converted into a cell mesh, and vice versa. For this specific protocol, the data is stored in a pandas DataFrame with a column for ‘frame’, which represents the frame number, ‘cell_id’, which represents the cell number in that frame and ‘mesh’, which is the cellular mesh structure.

## Key resources table

**Table:** 

REAGENT or RESOURCE	SOURCE	IDENTIFIER
**Bacterial and virus strains**		

*E. coli* MG1655	Guyer et al.^8^	(Ec)

**Chemicals, peptides, and recombinant proteins**		

Yeast extract	Oxoid	CAT#LP0021
Tryptone	Oxoid	CAT#LP042B
NaCl	Sigma-Aldrich	CAT#71380
Agar	BD	CAT#2038654
Agarose	Eurogentec	CAT#310526
Na_2_HPO_4_	VWR	CAT#1741
KH_2_PO_4_	Merck Life Science	CAT#1048731000
NH_4_Cl	Sigma-Aldrich	CAT#199970010
MgSO_4_	Sigma-Aldrich	CAT#M1880
CaCl_2_	Merck Life Science	CAT#1.02382.1000
L(+)-arabinose	Fisher Scientific	CAT#104980250
Glucose	Fisher Scientific	CAT#G/0450/65
Glycerol	Acros Organics	CAT#158920010
L-alanine	Fisher Scientific	CAT#451852500
Casamino acids	Fisher Scientific	CAT#12747109
Thiamine hydrochloride	Fisher Scientific	CAT#148991000
Vaseline	Merck Life Science	CAT#16415
Lanolin, anhydrous	Fisher Scientific	CAT#413005000
Paraffin	VWR	CAT#10048500

**Deposited data**		

MG1655 Images & Meshes	This paper; Mendeley Data	https://doi.org/10.17632/ynj2m6zt6x.1

**Software and algorithms**		

Python programming language	Python Software Foundation	https://www.python.org/downloads/
Anaconda	Anaconda, Inc.	https://www.anaconda.com/products/individual
Git	GitHub, Inc.	https://git-scm.com/downloads
Jupyter Notebook	Project Jupyter	https://jupyter.org/install
Notebooks to extract features, label data, and train SVM	This paper	https://github.com/Govers-Lab/Steemans_and_Govers_2023.git
NumPy	http://www.numpy.org	RRID: SCR_008633
pandas	https://pandas.pydata.org	RRID: SCR_018214
openCV	https://opencv.org/	RRID: SCR_015526
scikit-image	https://scikit-image.org/	RRID: SCR_021142
SciPy	http://www.scipy.org/	RRID: SCR_008058
scikit-learn	http://scikit-learn.org/	RRID: SCR_002577
Matplotlib	http://matplotlib.sourceforge.net	RRID: SCR_008624

**Other**		

Fisherbrand glass square coverslips	Fisher Scientific	CAT#12393138
Rogo Sampaic glass microscope slides	Fisher Scientific	CAT#11864782
Tape, temperature resistant (yellow, 25.4 mm)	VWR	CAT#817-1631
Gas burner	VWR	N/A
Immersion oil type F 30cc	Nikon	N/A

## Materials and equipment

**Table:** LB (Lennox) medium

Reagent	Amount
Yeast extract	2.5 g
Tryptone	5 g
NaCl	2.5 g
H_2_O	up to 500 mL
**Total**	**500 mL**

Autoclaved (121°C, 20 min) LB can be stored at room temperature (20°C–25°C) for multiple weeks. When you detect signs of contamination, do not use the medium and discard it using the appropriate procedures.

**Table:** LB-agar plates

Reagent	Amount
Yeast extract	2.5 g
Tryptone	5 g
NaCl	2.5 g
Agar	7.5 g
H_2_O	up to 500 mL
**Total**	**500 mL**

Poured LB-agar plates can be stored at 4°C for multiple weeks.

**Table:** 10× M9 salts

Reagent	Amount
Na_2_HPO_4_	60 g
KH_2_PO_4_	30 g
NaCl	5 g
NH_4_Cl	10 g
H_2_O	up to 1000 mL
**Total**	**1000 mL**

Autoclaved (121°C, 20 min) 10× M9 salts can be stored at room temperature (20°C–25°C) for multiple weeks.

**Table:** M9 medium + carbon source (L-arabinose, glucose, glycerol or L-alanine)

Reagent	Amount
10× M9 salts	100 mL
1 M MgSO_4_	2 mL
1 M CaCl_2_	0.1 mL
Carbon source (10%)	4 mL
H_2_O	893.9 mL
**Total**	**1000 mL**

Filter sterilized M9 medium + carbon source can be stored at room temperature (20°C–25°C) for several weeks. When you detect signs of contamination, do not use the medium and discard it using the appropriate procedures.

***Note:*** The M9 medium + carbon source can be supplemented with casamino acids (final concentration of 0.1%) and thiamine hydrochloride (final concentration of 1 μg/mL) to increase growth rate and yield.

**Table:** Agarose pads for microscopy (prepared in glass culture tubes)

Reagent	Amount
Medium/Buffer	3 mL
Agarose	0.045 g
**Total**	**3 mL**

Dissolve the agarose by gradually heating up the mixture with the flame of a Bunsen burner. Carefully remove the glass tube from the flame when the mixture starts to boil to avoid excessive evaporation or an overflow. Repeat until the agarose dissolves completely. This process typically takes approx. 2 min. As boiling is minimized, there is no need to account for evaporation of water during this process. For these agarose pads, either growth medium or a buffer can be used.

***Note:*** A glass tube with a solidified mixture of medium/buffer and agarose can be stored for one day at room temperature. To reuse, reheat the mixture with a Bunsen burner but exercise caution as pressure can build up from the bottom causing the solidified mixture to shoot upwards. Do not reheat the solidified mixture more than three times.

**Table:** VALAP Sealant^9^

Reagent	Amount
Vaseline	50 mL
Lanolin	50 mL
Paraffin	50 mL
**Total**	**150 mL**

After heating, VALAP can be kept for over a year at room temperature.

***Note:*** Heat the mixture on a hot plate (90°C) before each use. When it liquifies, the color should be golden yellow. Darker brown indicates overheating of the mixture.

### Microscopy

Phase contrast imaging was performed using a Nikon Eclipse Ti2-E inverted microscope, equipped with a CFI Plan Apo 1.45 NA DM Lambda 100× phase contrast oil objective, a Kinetix sCMOS camera (Teledyne Photometrics), and a built-in Perfect Focus system. The microscope was controlled by the NIS-Elements AR software.

## Step-by-step method details

### Bacterial growth and microscopy to generate training data

**Timing: 3 days**

In the context of this protocol, this step is not strictly required as one could use already existing or publicly available (segmented) image datasets (of bacterial cells). We also provide our own set of images and cell meshes together with this protocol (see our Mendeley data repository in the key resources table). This step describes the generation of snapshot images of wild-type *E. coli* cells sampled during exponential growth. For the training data, we used five different nutrient conditions that support a wide range of doubling times and cell sizes^1^: LB, M9 medium containing L-arabinose, casamino acids, and thiamine (M9LaraCAAT), M9 medium containing glucose (M9glu), M9 medium containing glycerol (M9gly), and M9 medium containing L-alanine (M9Lala).

1. Inoculation and growth.
  a. Streak *E. coli* from a 25% glycerol stock stored at -80°C onto an LB plate.
  b. Incubate the plate overnight at 37°C.
  c. Inoculate a single colony into a sterile tube containing 3 mL of one of the growth media. This step can be repeated for replicates and different nutrient conditions.
  d. Incubate at 37°C and 200 rpm for 20–24 h.
  e. Upon reaching stationary phase, dilute the preculture at least 10 000-fold into 3 mL of fresh growth medium.
  f. Incubate at 37°C and 200 rpm until the culture reaches an optical density at 600 nm (OD_600_) that corresponds to early exponential phase.

**Table 1:** All tested nutrient conditions and the approximate time it requires to reach an OD_600_ of 0.2 at 37°C after 10,000-fold dilution

Nutrient condition	Required time to OD_600_ of 0.2
LB	∼ 6 h
M9LaraCAAT	∼ 10 h
M9glu	∼ 18 h
M9gly	∼ 22 h
M9Lala	∼ 40 h

***Note:*** Typically, this is somewhere in between an OD_600_ of 0.1 and 0.3, but this depends on the nutrient condition and size of the cells. This way, the cultures are able to grow a large number of generations since stationary phase (≥ 8 generations) and are sampled before nutrient depletion and potential buildup of secondary metabolites. Table 1 contains the approximate time it takes to reach an OD_600_ of 0.2 in all five nutrient conditions (at 37°C) that were used in this protocol (troubleshooting 2).

***Alternatives:*** This methodology also works for cells sampled during other growth phases (e.g., lag or stationary phase).

***Note:*** To account for potential variations in growth between replicates or nutrient conditions, it is recommended to make multiple dilutions (e.g., 10 000-fold, 100 000-fold, and 200 000-fold) for each nutrient condition. This increases the likelihood of obtaining multiple cultures within the desired OD_600_ range when sampling for microscopy.

**Figure 2:**
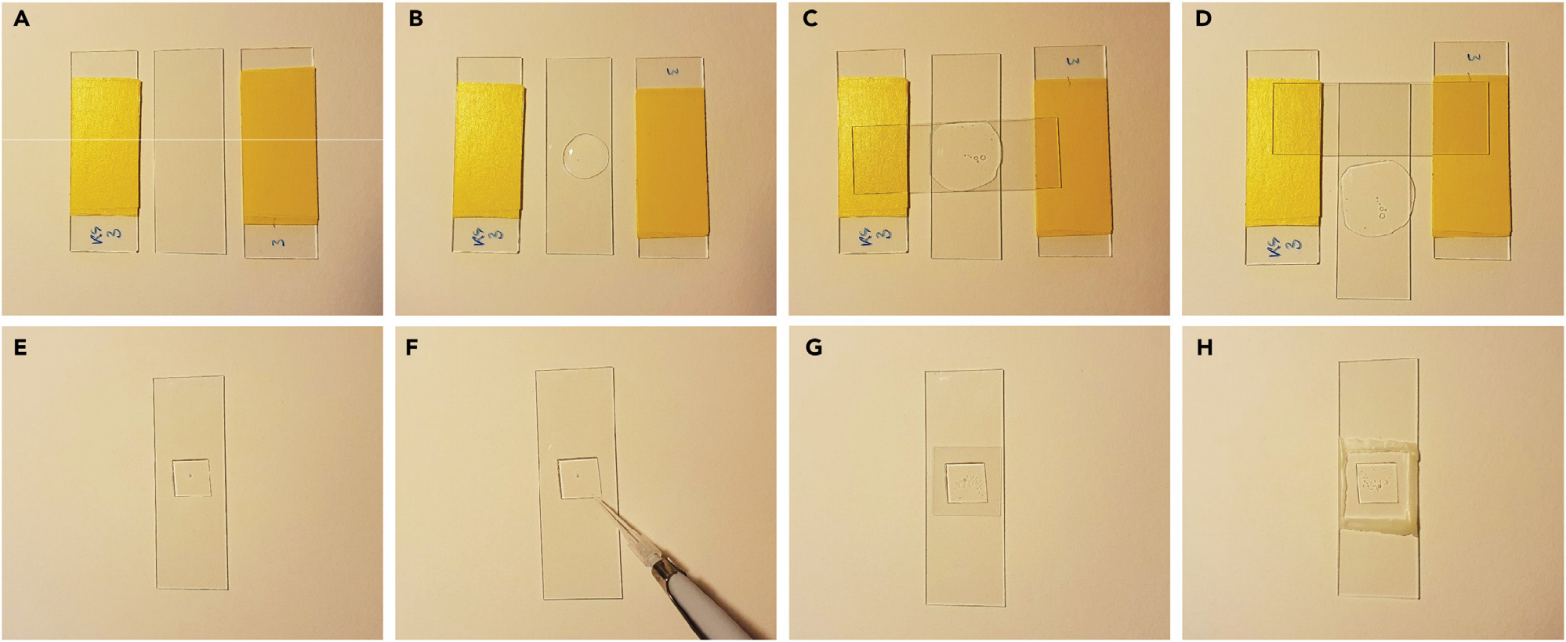
Preparation of a sample for microscopy All panels show different steps in the preparation of agarose pads for microscopy. (A) Three microscope slides, placed adjacent to each other, with three layers of tape on the outer two slides. (B) 300 μL of a heated mixture of medium/buffer and agarose is spotted on the middle slide. (C) A fourth microscope slide is placed on the droplet, perpendicular to the other three slides. (D) After solidifying (∼15 min) and trimming of the edges using a razor blade, the middle slide is gently moved downwards in a single fluent motion. (E) Uneven edges were removed from the agarose pad using a razor blade. (F) 0.5 μL of cell culture was spotted on the agarose pad and allowed to dry. (G) Placement of a cover glass over the agarose pad. (H) The cover glass was sealed using pre-heated VALAP and a cotton swab.
  b. Gradually heat up a glass culture tube containing medium/buffer and agarose powder with a Bunsen burner. Carefully move the glass tube in and out of the flame as the liquid starts to boil. Do this until the agarose dissolves (Figure 3).

2. Prepare VALAP Sealant.
  a. Turn on the hot plate at 90°C.
  b. Heat up the VALAP in a small glass cylinder until it liquifies.
3. Prepare agarose pad.
  a. Take four microscope slides. Put three layers of tape on one side of two microscope slides. Place a third glass slide in between the two slides with tape (Figure 2A).

**Figure 3:**
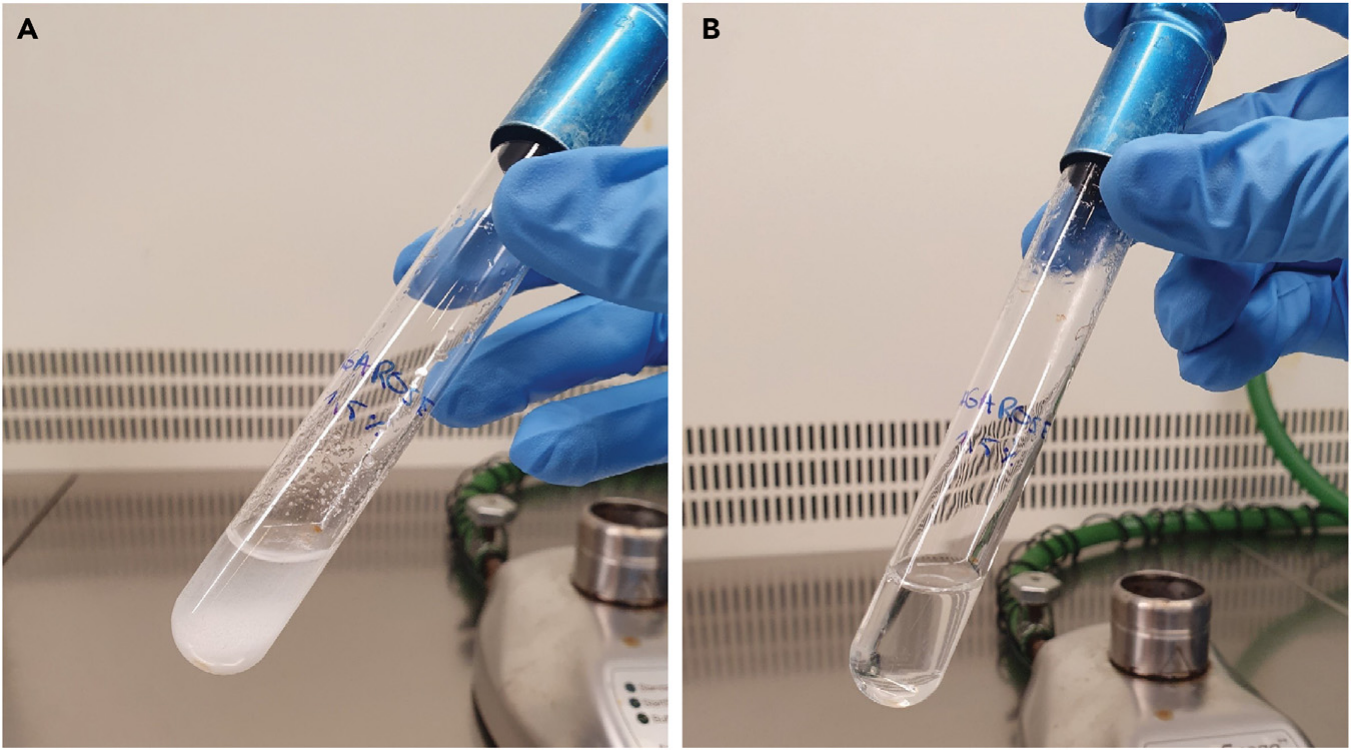
Dissolving the mixture of medium/buffer and agarose with a Bunsen burner Panel A is before, panel B after heating.
  c. Pipet 300 μL of the hot mixture onto the middle slide without tape (Figure 2B).
  d. Take the fourth microscope slide and place it on top of the liquid, perpendicular to the other three slides, so that the slide is supported by a slide with tape on each side. The tape serves as a spacer, and determines the thickness of the agarose pad (Figure 2C).
  e. Let the agarose solidify for ∼15 min.

***Note:*** To ensure consistent thickness of your pad, do this on a flat and clean surface. Also, it is recommended to not put the fourth slide down too gently as this leads to an uneven and tilted surface of the pad. One way to do this is by placing one side of the fourth slide on a slide with tape almost vertically and allowing it to drop onto the other slide with tape, over the slide with the agarose mixture. Do not touch the slides while the mixture is solidifying. The quality of an agarose pad can only be assessed during imaging. An ideal situation is a flat and uniform surface where all visible cells are in the same focal plane. Uneven pads typically lead to situations where certain cells are in focus and others are not (within the same image).

**Figure 4:**
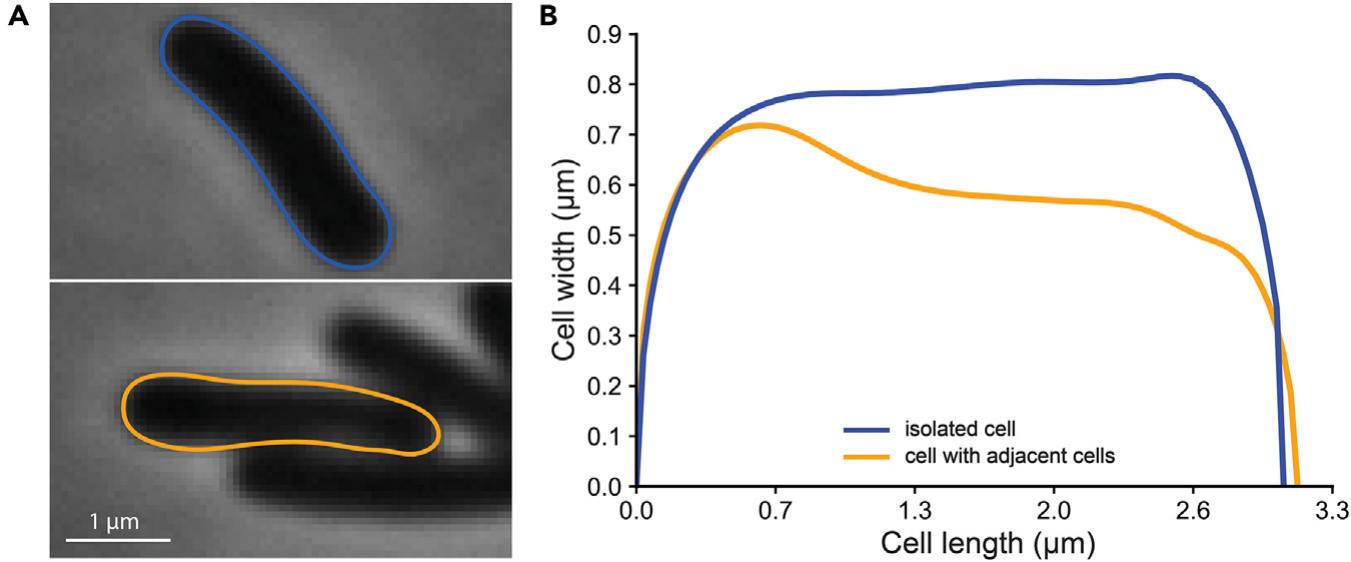
Adjacent cells affect cell morphological measurements (A) Representative phase contrast image and contour of an isolated cell (top) and a cell with multiple adjacent cells (bottom). (B) Line plot of cell width along the cell length of cells shown in panel A.
  c. Use a software package of your choice to detect individual bacterial cells in the obtained phase contrast images.
    ***Note:*** Various segmentation software and algorithms have been developed in recent years that enable the identification of individual bacterial cells in phase contrast microscopy images (e.g., Oufti,^7^ MicrobeJ,^2^ Omnipose,^6^ DeLTA2.0^5^, SuperSegger^10^). While this protocol focuses on the cell mesh structure that is generated by Oufti,^7^ any cell contour coordinates, independently of how they were obtained, can be converted into a cell mesh.
    ***Note:*** Our imaging data and corresponding cell meshes can be found in our Mendeley data repository (key resources table).

4. Sample preparation (troubleshooting 3).
  a. Retrieve the cultures from the shaking incubator when they reach the required OD_600_.
  b. Remove excess agarose that might have escaped the overlap region between the slides with a single edge razor blade.
  c. While holding the fourth microscope slide, gently push or pull the middle microscope slide up- or downwards in a single fluent motion to remove it from under the fourth microscope slide (Figure 2D).
  d. Take a razor blade and cut the uneven edges from the pad (Figure 2E).
  e. Spot 0.5 μL of the culture on the pad. Multiple spots can be combined onto a single pad, but care must be taken to ensure that they are placed sufficiently far from each other to prevent droplets from merging (Figure 2F).
    ***Note:*** Ensure that the cells are spotted away from the edges of the agarose pad. This prevents the VALAP from touching the microscope objective and interfering with the imaging.
  f. Let the spot dry.
  g. Take a cover glass, appropriate for the imaging setup and objective to be used, that is larger than the agarose pad and place it on the agarose pad (Figure 2G).
  h. Seal the cover glass with the pre-heated VALAP sealant mixture using a cotton swab (Figure 2H). Gently dip the cotton swap in the liquid VALAP and then streak it in a straight line along an edge of the cover glass. Do this for each side of the cover glass so that it is sealed completely.
    ***Note:*** Avoid getting VALAP on top of the cover glass, especially in the region where cells have been spotted.
  i. The sample is now ready to be imaged. The desired time to perform imaging of live cells is within 5–10 min after spotting the cells on the pad.
5. Microscopy (troubleshooting 4) and image analysis
  a. Use the objective of your choice.
    ***Note:*** Typically, either a 60× or 100× phase contrast oil objective is used for bacterial samples. For this protocol, we used a 100× phase contrast oil objective (Numerical Aperture = 1.45). Most available segmentation software or algorithms work across different magnifications. The same is true for an SVM model, as long as images acquired using different magnifications are included in the training dataset.
  b. Choose different XY positions and take the snapshots.
    ***Note:****Try to capture as many individual, non-touching cells as possible. This is because cell morphological measurements, and especially width measurements, are affected by cells lying adjacent to other cells (*Figure 4*). We observed this effect independent of the segmentation algorithm that was used. For this reason, also avoid positions close to the border of the culture spot, as these typically contain large and dense clumps of cells. Cultures with an OD*_*600*_*between 0.1 and 0.3 typically have a good density and should have a nice spread under the microscope. For this protocol, we acquired images with an exposure time of 200 ms, no binning, and a bit depth of 16-bit.*

### Feature extraction

**Timing: 15 min**

In this step, cellular features are extracted from the cell meshes and phase contrast images, using the ‘feature_extraction.ipynb’ notebook. This will result in a DataFrame with 26 features that, together with the frame number, cell ID and cell contour, are exported in a pickle file. The feature extraction script can deal with segmentation results obtained from various sources and/or algorithms, as long as these results come in the form of cell meshes (described above). To test the notebook, you can use the cell meshes and images provided in our Mendeley data repository (key resources table).

6. Activate the virtual environment (svm_env) and open Jupyter Notebook.
7. Open the feature_extraction.ipynb file inside the ‘Notebooks’ folder of the Steemans_and_Govers_2023 repository.
8. Run every code cell of the notebook in the correct order:
  a. **Import necessary libraries**: run this code cell to import the required Python libraries (troubleshooting 1).
  b. **Load image and mesh data**: to load raw images, provide the path to the folder where the images are located. Images should be stored as separate TIF files in this folder. To load the cell mesh data, specify both the path and the file name where the mesh data is stored.
  c. **Load functions**: instead of adding a separate helper function file, we opted to include the functions that calculate the features in the notebook itself. Running this code cell will load in the functions that are used in the subsequent code cell of the notebook.
  d. **Extract features**: running this code cell will extract the features from the cell meshes.
    ***Note:*** After feature extraction, the first five rows are displayed underneath the code cell. This serves as an initial validation step that allows a quick assessment of the calculated feature values, and whether or not these were calculated successfully.
  e. **Save DataFrame:** save the DataFrame to a specific folder by changing the file_path. Preferably, the name of the pickle file should end with ‘svm_features.pkl’.

### Labeling cell detections

**Timing: 1–2 days**

In order to label cell detections as ‘good’ or ‘bad’, the DataFrame generated in the previous section (containing the frame number, cell id, cell contour, and cell features) is required. After completion of the current section, the DataFrame will have an extra column ‘label’, where a value of 1 corresponds to a correct cell detection, 0 to an incorrect cell detection, and NaN (Not a Number) to an ambiguous cell detection. The duration of this step can vary depending on the desired size of the training dataset. In our experience, 20 000 cells can be labeled in less than 12 h. This part of the protocol can be tested using our example data located in the Feature_Data folder of the Steemans_and_Govers_2023 GitHub repository.

**Figure 5:**
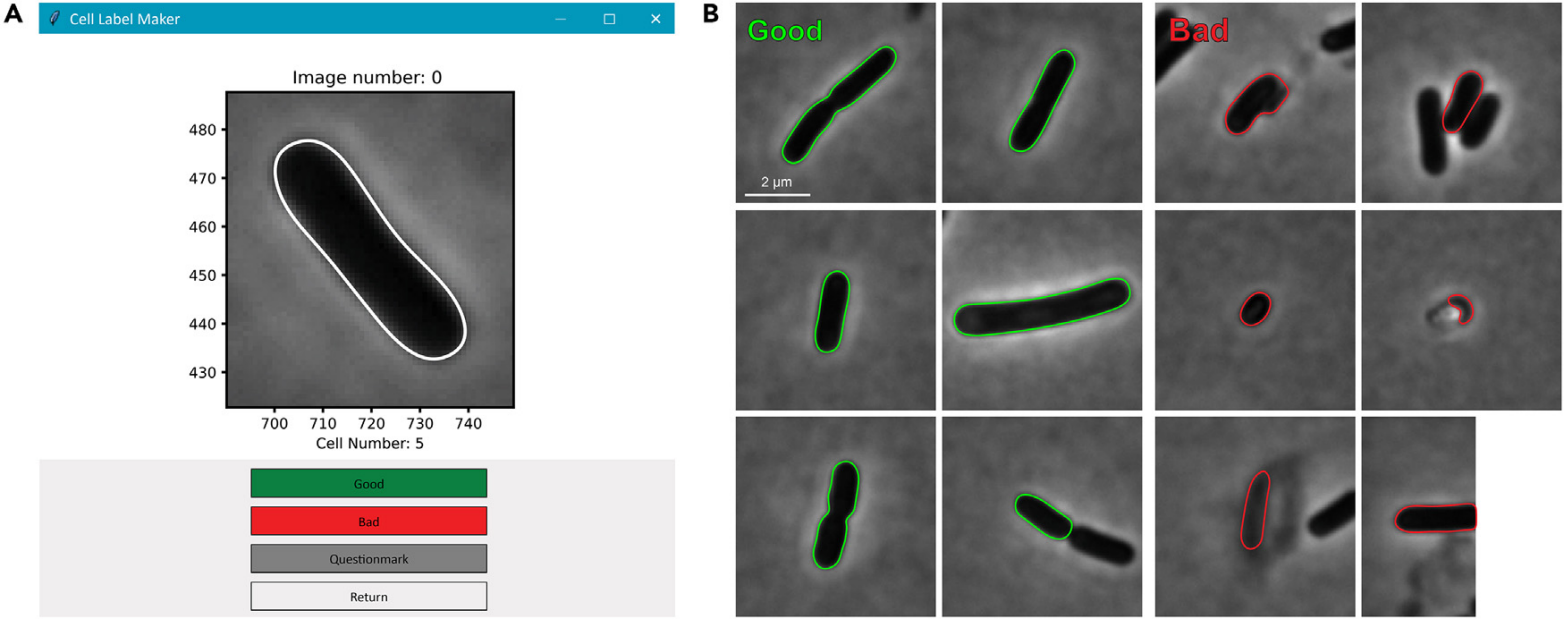
Graphical user interface (GUI) for labeling cell detections and examples of ‘Good’ and ‘Bad’ cell detections (A) Image of the GUI that enables the user to label individual cell detections as 'Good', 'Bad', or 'Questionmark'. The ‘Return’ button allows the user to go back to the previous cell and overwrite a past assessment. (B) Representative examples of cell detections that are considered ‘Good’ (green) and ‘Bad’ (red).
  e. To conclude the labeling process, simply close the window displaying the GUI. Upon closure, the current frame and cell ID will be printed, indicating the progress of the labeling process. All cells up to the current cell will have been labeled, with the corresponding information stored in the DataFrame.
  f. **Save DataFrame:** save the DataFrame to a specific folder by changing the file_path.
    ***Note:*** In the event of an interruption or the need to stop the labeling process and restart at a later time, it is possible to adjust the code by modifying the current frame and cell ID values. By updating these variables to the desired starting point, the labeling can be resumed from the specified position. If the interruption occurs, it is essential to first save the DataFrame and then reload that saved file to ensure that no labeling information is lost.

9. Open the labeling.ipynb file inside the ‘Notebooks’ folder of the Steemans_and_Govers_2023 repository.
10. Run every code cell in the notebook in the correct order:
  a. **Import necessary libraries:** run this code cell to import the required Python libraries (troubleshooting 1).
  b. **Load functions:** to load helper functions that are used in subsequent code cells.
  c. **Load data:** both the feature data and the images are loaded here. Change the image_path and svm_data_path accordingly to ensure loading of the correct files.
  d. **Initialize GUI and start labeling** (troubleshooting 5): upon running this code cell, a graphical user interface (GUI) will appear (Figure 5A), presenting four distinct buttons: 'Good', 'Bad', 'Questionmark', and 'Return'. Each cell and its corresponding cell contour will be loaded into the GUI, after which the user can assess the cell detection. If the cell was detected correctly, click the ‘Good’ button. If the cell was detected incorrectly (e.g., only part of the cell was detected or a neighboring cell affects the cell contour), click the ‘Bad’ button. In case of doubt, click the ‘Questionmark’ button. Upon clicking, the next cell and its corresponding detection will be displayed for their assessment. The ‘Return’ button allows the user to go back to the previous cell and overwrite a past assessment.
    ***Note:***Figure 5B contains several examples of ‘Good’ and ‘Bad’ cell detections. To train the SVM model to discard cells with adjacent cells, these cells should be identified as ‘Bad’ cell detections during the labeling process.

### Train and validate a support vector machine

**Timing: 30 min**

This step involves leveraging the labeled feature data to train an SVM with sufficient discriminatory power. The notebook we used pre-processes the data, trains multiple SVMs with different hyperparameters, chooses the model with the best parameters for deployment and evaluates its performance on unseen test data. In order to test this notebook, users can use our labeled data in the Labeled_Data folder of the Steemans_and_Govers_2023 GitHub repository.

11. Open the train_svm.ipynb file inside the ‘Notebooks’ folder of the Steemans_and_Govers_2023 repository.
12. Run every code cell in the notebook in the correct order:
  a. Load necessary libraries.
  b. **Load data:** all labeled feature data is required for this step. Provide the path to directory where the svm_feature pickle files are located. Ensure that the glob algorithm searches for the files with the labeled feature data (and not the raw feature data). All loaded DataFrames will be merged and used for further pre-processing.
  c. Data preparation:
    i. **Removal of NaNs:** NaN values could hamper the downstream analyses and thus need to be removed.
    ii. **Split data & class proportions** (troubleshooting 6): this part of the code extracts the feature columns from the DataFrame and splits the data in a test- and training set. To change the size of the test set, you can adjust the parameter test_size in the method train_test_split().
      ***Note:*** Typical train-test ratios include 80-20, 67-33 and 50-50, but no single perfect ratio exists. The choice of split ratio is often context-dependent and needs to factor in the computational cost for training and testing the model, while ensuring that both classes of cell detections (good and bad) are significantly represented in both the training and test sets. Also important to consider are the class proportions (i.e., the ratio of good vs. bad cell detections). If class imbalances exist (i.e., less than 1% of detected cells are bad cell detections), other training approaches might be more appropriate as the performance of a trained model can drop significantly.^11^ In our experience, this does not often occur in bacterial microscopy data. For our example data, we used a 50-50 test-train ratio as the dataset is of significant size and the positive-negative class ratio is relatively balanced (∼63%–37%).
    iii. **Feature correlations:** generation of a correlation heatmap of features to examine potential feature redundancies.
      ***Note:*** Multicollinearity can be a reason to eliminate a feature when training an SVM model. However, this by itself should not be the sole criterion for eliminating a feature. Context and domain knowledge should also be considered. Features that display strong correlations could still provide unique or complementary information to the model. Therefore, it is essential to interpret and analyze the relationship between features further before eliminating them.
  d. **Train SVM**: in order to find the optimal hyperparameters to train the SVM, a 5-fold cross-validation is performed with a linear or non-linear kernel (radial basis function, RBF kernel), a range of C values (100, 1000, 10 000) and a range of gamma values (0.001, 0.01, 0.1). K-fold cross-validation is a technique for tuning these hyperparameters that involves resampling by dividing the training data into k subsets. During each iteration, the model trains on k-1 subsets of the train set and evaluates on the remaining subset. This process is repeated k times, and the hyperparameters of the most successful model are selected for validating on the test data (troubleshooting 7).
    i. **With Nyström method**: the Nyström method is a powerful technique used to approximate kernel functions, which play a key role in reducing the computational complexity associated with solving optimization problems. By leveraging a subset of representative points from the dataset, the Nyström method constructs a low-rank approximation of the kernel matrix. This approximation captures the essential information of the original kernel matrix while significantly reducing the number of computations required. The Nyström method is particularly beneficial when dealing with large datasets (> 5000 training data points), as it enables efficient and scalable solutions without sacrificing much accuracy.^12^
    ii. **Without Nyström method:** for small training datasets (< 5000 training data points), the optimal hyperplane that separates the two classes can be efficiently determined without the Nyström method.
      ***Note:*** If you want to apply the Nyström method with a linear kernel, change ‘rbf’ to ‘linear’ in the code cell ‘Train SVM with Nyström method’.
  e. **Validation with receiver operating characteristic (ROC) curve**: the area under the ROC curve (AUC) is commonly used as a measure of the classifier’s overall performance. The AUC value ranges from 0 to 1, with a higher value indicating better classifier performance. An AUC of 1 represents a perfect classifier that separates the classes without error, while an AUC of 0.5 indicates a random or ineffective classifier that performs no better than chance.^13^
    ***Note:*** For validation with an ROC curve the probability argument in Pipeline() during training has to be set to 'True'.
  f. **Confusion matrix:** running this code cell will construct a confusion matrix from which performance metrics are calculated (accuracy, precision, recall, specificity and F1 score). For our application, it is particularly important to minimize false positive cases as these represent incorrect cell detections that would be included in further analysis.
  g. **Evaluation of bias:** in order to evaluate whether the model introduces bias, the ratios between the mean values of each feature for the manually curated and model evaluated datasets are calculated. If a ratio deviates from 1, the model introduces a bias for that particular feature and the use of this model should be avoided.
  h. **Save Model**: the best model can be saved by running this cell. Specify the path (including the name) where you want to save the model.

## Expected outcomes

Ultimately, this protocol should yield a trained SVM classifier that determines whether cell detections of individual bacterial cells are suitable for further analysis or not with a reasonable accuracy (90%–100%). This model can be used to evaluate new cell detections by extracting features from the meshes using the feature_extraction.ipynb notebook, loading the best performing model and classifying cell detections using the newly created feature DataFrame (excluding the non-feature columns).

Together, our protocol provides a comprehensive overview of the different steps that are required for training an SVM model (from generating training data, to extracting relevant features, labeling cell detections, training an SVM model and assessing its performance), accessible to users with limited programming experience. This is done to encourage researchers to use machine learning for automating the cumbersome manual curation of cell detections. Such automation efforts are important, as bioimage data is becoming increasingly more common and generating such image datasets (with hundreds of individual cells per frames and several frames per experiment/strain) is becoming increasingly less difficult.

## Quantification and statistical analysis

Overview of the cellular features that are extracted from cell meshes and phase contrast images, along with an explanation of how they are calculated.

**Cell length (μm):** individual step lengths (between each segment of the cell mesh) are determined by computing the distance between the midpoints of the lines formed by the coordinates (x1, y1) and (x2, y2). The calculated step lengths are subsequently summed to obtain the total cell length.

**Cell width (μm):** the cell width is computed as the mean of the top one-third of the segment widths (i.e., are the distances between coordinates (x1, y1) and (x2, y2)).

**Cell area (μm**^**2**^**):** the pixel area is computed based on the contour that outlines the cell using shapely.Polygon().

**Cell volume (μm³):** each cell segment is assumed to be a cylinder, and the volume of each cell segment is computed using the trapezoidal rule and summed up to obtain the total volume of the cell.

**Cell surface area (μm**^**2**^**):** each cell segment is assumed to be a cylinder, and the surface area is calculated as $2\pi *\left(\frac{width}{2}\right)*step\_length$. Individual surface areas of each cell segment are summed to obtain the total surface area.

**Max, min and mean contour curvature:** the curvature characteristics are computed using the gradient and the second derivative of the contour coordinates.

**Cell perimeter (μm):** the perimeter is calculated by summing up the distances between two consecutive points of the (subpixel) cell contour. From the cell perimeter, other features such as circularity, compactness, and sphericity are calculated. **Cell circularity:**

$$\frac{4\pi *area}{perimeter^{2}}$$

**Cell compactness:**

$$\frac{perimeter^{2}}{area}$$

**Cell sphericity:**

$$\frac{1.5\pi {\left(\frac{perimeter}{2\pi }\right)}^{1.5}}{area}$$

**Total phaco intensity (A.U.):** the sum of all phase contrast pixel intensity values inside the cell contour.

***Note:*** the phase contrast image is inverted, and a background subtraction is performed by deducting the mean intensity value outside all cell segmentation masks from the image.

**Max and mean phaco intensity (A.U.):** Maximum and mean of the phase contrast pixel intensity values inside the cell contour.

**Phaco contour intensity peaks (A.U.):** The number of peaks in the phase contrast intensity along the cell contour, determined using the find_peaks() method from the SciPy library.

**Max, min and mean contour intensity (A.U.):** The maximum, minimum and mean values of the phase contrast intensity along the cell contour.

**Max and mean contour intensity variability:** The rolling window standard deviation is computed for the contour intensity values using a window size of 10. This captures fluctuations in contour intensity along the contour, and calculates both the maximum and mean values.

**Midline intensity skewness:** The skewness of the distribution of phase contrast intensity values along the cell midline.

**Midline intensity kurtosis:** The kurtosis of the distribution of phase contrast intensity values along the cell midline.

**Max and mean expanded contour intensity (A.U.):** The contour is expanded by 2 pixels and the maximum and mean phase contrast values along the expanded contour are then calculated.

**Mean cell edge gradient (A.U.):** This feature uses both an expanded and an eroded cell contour. The intensity values along these contours are subtracted from each other and averaged to calculate the mean gradient over the cell contour.

## Limitations

Any model is only as good as the data it has been trained on. As such, SVM models trained on a specific species could underperform on other species. Especially taking into account the wide range of cell morphologies present in the bacterial domain,^14^ retraining of the model or the design of novel features to extract from the cell meshes might be required for optimal SVM performance.

This protocol is mainly intended for the analysis of microscopic snapshots of individual bacterial cells. These static images allow a detailed quantitative characterization of cell morphology and subcellular organization (when combined with fluorescent dyes and/or labels). Although the methodology could be expanded to the analyses of time-lapse images (where individual cells are tracked over time as they grow and divide), other factors should then also be considered (e.g., tracking cells between frames and determining division events).

## Troubleshooting

### Problem 1

Before you begin, step 2.

The module cannot be found or other Python library-related errors occur while running the code.

### Potential solution

• The module was not installed properly. Create a separate code cell and run:

>pip install <name of module>

This will update the previous version to the most recent version.

• In certain cases, compatibility issues may arise when installing a package using pip, particularly if the package version is not aligned with your operating system or other dependencies within your environment. To solve this problem, alternative channels (i.e., conda or conda forge) can be used to install the necessary packages. Ensure that the correct package versions are installed to avoid dependency issues.

### Problem 2

Bacterial growth and microscopy to generate training data, step 1f.

The cell culture is overgrown (i.e., exceeds the required OD_600_ range).

### Potential solution

Dilute the culture sufficiently in a glass tube containing 3 mL of fresh medium, and incubate again until it reaches the required OD_600_. Upon redilution, aim to give the culture at least 4–5 generations before reaching the required OD_600_.

### Problem 3

Bacterial growth and microscopy to generate training data, step 4.

The density of cells is too high on the agarose pad, resulting in a limited amount of single, non-neighboring cells. This can be a problem as the training of an SVM model requires sufficient positive and negative data.

### Potential solution

Dilute the culture sample before spotting on a new agarose pad.

### Problem 4

Bacterial growth and microscopy to generate training data, step 5.

The agarose pad has granular artefacts that could hamper segmentation and lead to a class imbalance with an underrepresentation of positive cases.

### Potential solution

Granular artefacts can be the result of poor dissolution of the agarose during preparation of the agarose pad. Reheat the agarose mixture of agarose and medium/buffer again, while ensuring all agarose particles are completely dissolved, and make a new pad to spot cells on. Otherwise, start with a fresh batch of agarose and medium/buffer.

### Problem 5

Labeling cell detections, step 10d.

Assignment of a label to a cell detection that balances between a 'good' and 'bad' cell.

### Potential solution

The task of labeling can be challenging and is undeniably prone to personal bias, as different people might have different opinions on different cell detections. Regardless of which label is ultimately assigned to an individual cell, it is crucial to maintain a certain consistency throughout the labeling process. This is important for preventing the introduction of biases that could potentially impact the model’s performance. If uncertainty remains, label the cell as ‘Questionmark’, which will lead to its exclusion during training of the model.

### Problem 6

Train and validate support vector machine, step 12c.

An imbalance exists among the classes, potentially leading to a bias of the SVM model towards the majority class. Moreover, the model’s generalization capability could be compromised, causing it to struggle in detecting instances from the minority class. In these cases, it is important to keep in mind that a model that is biased towards the majority class could still attain a seemingly impressive accuracy, yet demonstrate a subpar performance on the minority class.

### Potential solution

Class imbalances are a common problem in the field of machine learning. However, persistent efforts have led to the development of multiple methods aimed at effectively addressing this issue.^11^ Here, we elaborate on two popular solutions to this problem:

• SMOTE (or synthetic minority over-sampling technique) is an approach that creates new synthetic instances of the minority class in order to improve the model’s general performance.^15^ The Python library ‘imbalanced-learn’ provides the necessary tools to perform SMOTE.
• Cost-sensitive training assigns a cost to prediction errors during training. This is often translated into setting a high cost for misclassifications of the minority class.^16^ In order to apply this concept, change the ‘class_weight’ argument in the SVC method. Note that ‘class_weight’ is considered a hyperparameter that requires tuning.^17^

### Problem 7

Train and validate support vector machine, step 12d and 12g.

The SVM classifier performs suboptimal (< 90% accuracy) or introduces a bias for certain features.

### Potential solution

• Use a different range of hyperparameters in the grid search (i.e., 0.1, 1, 10 instead of 100, 1000, 10 000 for the C hyperparameter).
• Explore other kernel functions (e.g., polynomial or Laplacian).
• An underperforming model could also be a consequence of the features not having sufficient discriminatory power. In this case, the design and calculation of different cellular features is required.
• Train another type of machine learning model. Potential alternatives to SVMs include Random Forest,^18^ K-nearest neighbors (KNN)^19^ or Adaptive Boosting (AdaBoost).^20^

## Resource availability

### Lead contact

Further information and requests for resources and reagents should be directed to and will be fulfilled by the lead contact, Sander K. Govers (sander.govers@kuleuven.be).

### Technical contact

Further questions about the technical specifics of performing the protocol should be directed to and will be fullfiled by the technical contact, Sander K. Govers (sander.govers@kuleuven.be).

### Materials availability

This study did not generate new unique reagents.

### Data and code availability

The code and data generated during this study are available at https://github.com/Govers-Lab/Steemans_and_Govers_2023.git.

The imaging data generated in this study is available at Mendeley Data: https://doi.org/10.17632/ynj2m6zt6x.1.
